# Protein Phosphatase 1 Dephosphorylates Profilin-1 at Ser-137

**DOI:** 10.1371/journal.pone.0032802

**Published:** 2012-03-30

**Authors:** Jieya Shao, Marc I. Diamond

**Affiliations:** Department of Neurology, School of Medicine, Washington University, St. Louis, Missouri, United States of America; Vanderbilt University Medical Center, United States of America

## Abstract

Profilin-1 (PFN1) plays an important role in the control of actin dynamics, and could represent an important therapeutic target in several diseases. We previously identified PFN1 as a huntingtin aggregation inhibitor, and others have implicated it as a tumor-suppressor. Rho-associated kinase (ROCK) directly phosphorylates PFN1 at Ser-137 to prevent its binding to polyproline sequences. This negatively regulates its anti-aggregation activity. However, the phosphatase that dephosphorylates PFN1 at Ser-137, and thus activates it, is unknown. Using a phospho-specific antibody against Ser-137 of PFN1, we characterized PFN1 dephosphorylation in cultured cells based on immunocytochemistry and a quantitative plate reader-based assay. Both okadaic acid and endothall increased pS137-PFN1 levels at concentrations more consistent with their known IC_50_s for protein phosphatase 1 (PP1) than protein phosphatase 2A (PP2A). Knockdown of the catalytic subunit of PP1 (PP1Cα), but not PP2A (PP2ACα), increased pS137-PFN1 levels. PP1Cα binds PFN1 in cultured cells, and this interaction was increased by a phosphomimetic mutation of PFN1 at Ser-137 (S137D). Together, these data define PP1 as the principal phosphatase for Ser-137 of PFN1, and provide mechanistic insights into PFN1 regulation by phosphorylation.

## Introduction

Profilins are small actin-binding proteins that are essential for all eukaryotic cells. They play a role in many cellular processes including cell motility, cytokinesis, gene transcription, endocytosis and neuronal plasticity [1], [2], [3], [4]. These activities depend on their interactions with three major cellular ligands: globular actin (G-actin), polyproline-containing proteins, and phosphatidylinositols (e.g. phosphatidylinositol 4,5*-*bisphosphate, PIP2). In mammals, four profilin isoforms have been identified, each encoded by a distinct gene. Profilin-1 (PFN1) and profilin-2a (PFN2a), the classic “somatic” isoforms, are 65% identical in amino acid sequence, and highly conserved in 3D structure [3]. PFN1 is ubiquitously expressed, while PFN2a, the major splice isoform of PFN2, is preferentially enriched in the brain [3]. Two testis-specific profilins, PFN3 and PFN4, are recently described, and differ substantially from PFN1 and PFN2a in their primary sequences [5]. Both PFN3 and PFN4 bind G-actin with lower affinity than PFN1 and PFN2a. PFN3 binds polyproline-ligands poorly, and PFN4 completely lacks this activity [5].

Recent studies have linked profilins, in particular PFN1, to several human diseases. Our prior work identified PFN1 as an inhibitor of huntingtin aggregation, suggesting a role in the pathogenesis of Huntington Disease (HD). The anti-aggregation activity of PFN1 depends on its binding to both G-actin and a polyproline tract within huntingtin [6]. PFN1 is also a putative tumor suppressor, and has been shown to inhibit tumor cell growth and metastasis in several cancer models [7], [8], [9], [10], [11], [12].

Despite our detailed knowledge of profilin's structure and functions, we know little about how its cellular activities are regulated. PFN1 is a phospho-protein *in vivo* [13], [14], suggesting that its activities could be regulated by phosphorylation. We have previously determined that Ser-137 of PFN1 is a *bona fide* phosphorylation site for the Rho-associated kinase ROCK [6]. Ser-137 lies within the polyproline-binding site of PFN1. Mimicking phosphorylation at this site abolishes PFN1's binding to huntingtin, and inhibits its activity as an aggregation suppressor [6]. To our knowledge, this was the first study to link a specific phosphorylation event to defined cellular functions of PFN1, and to demonstrate that PFN1 activity is regulated. While our prior work identified ROCK as an upstream kinase for Ser-137, it left uncertain which phosphatase mediates dephosphorylation of this site. By exploiting a highly sensitive and specific PFN1 antibody against pSer-137, we now provide pharmacological, genetic and biochemical evidence that protein phosphatase-1 (PP1) exists in the same protein complex with PFN1 and dephosphorylates Ser-137.

## Results

### P3490 specifically recognizes pS137-PFN1 *in situ*

**Figure 1:**
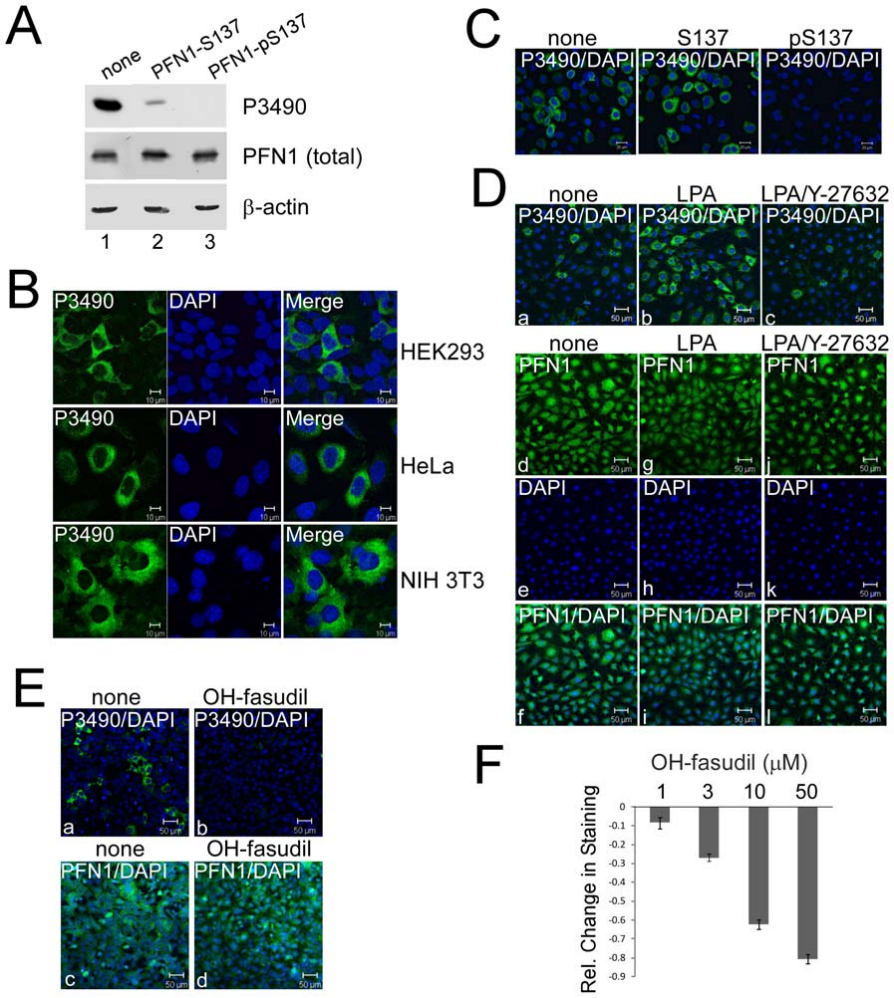
*In situ* staining of mammalian cells by pSer-137-PFN1 antibody P3490 is responsive to RhoA/ROCK signaling. *A*, Equal amounts of HEK293 cell lysate were separated on SDS-PAGE, and individual lanes were blotted with P3490 that was pre-incubated with no peptide (Lane 1), or PFN1 peptides containing unphosphorylated (Lane 2) or phosphorylated Ser-137 (Lane 3). PFN1-pS137 peptide completely blocked the binding of P3490 to PFN1. Unphosphorylated PFN1-S137 peptide blocked ∼90% binding vs. the no peptide control. Total PFN1 (on a separate and identically loaded gel) and actin levels were confirmed as being equal across all lanes. *B*, P3490 stains the cytoplasm of multiple cell lines in a heterogeneous fashion, including HEK293, HeLa and NIH 3T3 (in green). DAPI was used as counterstain (in blue). *C*, Immunostaining of HEK293 cells with P3490 (green) was unaffected by pre-incubation with the unphosphorylated PFN1-S137 peptide, but was completely inhibited by the phosphorylated PFN1-pS137 peptide. DAPI staining is shown in blue. *D*, NIH 3T3 cells were serum-starved for 16 hr in the absence or presence of 50 µM Y-27632, followed by treatment with 10 µM Lysophosphatidic acid (LPA) for 60 min. Cells were immunostained with P3490 (green) and counterstained with DAPI (blue) (a–c). LPA increased P3490 staining, which was blocked by the pretreatment with Y-27632. Total PFN1 levels were unaffected by these treatments as indicated by immunostaining with a generic PFN1 antibody (PFN1 in green and DAPI in blue) (d–l). *E*, HEK293 cells were treated with or without 50 µM hydroxyfasudil (OH-fasudil) for 2 hr, followed by immunostaining with P3490 (a–b) or the generic PFN1 antibody (c–d) (both in green) and counterstaining with DAPI (blue). Nearly all cells treated with OH-fasudil became P3490-negative without changes in their total PFN1 levels. *F*, HEK293 cells grown in a 96-well plate were treated for 24 hr with increasing concentrations of OH-fasudil (1, 3, 10, 50 µM), immunostained with P3490 and Alexa Fluor®488-labeled secondary antibody, and counterstained with DAPI. Fluorescence intensity was quantified on a fluorescence plate reader. P3490 staining was normalized vs. DAPI to control for cell numbers, and the effects of OH-fasudil were calculated as relative values as compared to the untreated cells. OH-fasudil reduced P3490 staining dose-dependently. Error bars represent the standard error of the mean (SEM). Data are mean ± SEM of three independent experiments.

We previously generated a polyclonal phospho-specific antibody against pSer-137 of PFN1 (P3490). Using this antibody, we previously determined by Western blot (WB) that pS137-PFN1 levels in cultured cells are regulated by RhoA/ROCK signaling. The RhoA activator lysophosphatidic acid (LPA) mildly increased pS137-PFN1 levels in cultured cells, which was blocked by the ROCK inhibitor Y-27632 [6]. To directly characterize the phospho-specificity of this antibody, we pre-incubated P3490 with antigenic peptides containing (pS137) or lacking a phosphate on Ser-137 (S137), followed by WB to determine if its reactivity with cellular PFN1 was affected. The pS137-peptide completely blocked P3490 binding to PFN1 (Fig. 1A) as expected. However, the non-phospho S137-peptide effectively blocked 90% of the PFN1 signal versus the no peptide control (Fig. 1A). Thus, P3490 contains pan-antibodies that react with unphosphorylated PFN1, and the bulk of the observed PFN1 signal likely represents cross-reactivity with unphosphorylated PFN1 molecules that outnumber those of phospho-PFN1. This limits the fidelity of P3490 to accurately report pS137-PFN1 levels via WB.

Due to the limitation of P3490 associated with WB, we tested if it can detect pS137-PFN1 *in situ* by immunocytochemistry. P3490 heterogeneously stained several cell lines (predominantly the cytoplasm), i.e. not all cells were positive at the same time (Fig. 1B). The cause for this staining pattern of P3490 is unclear, but could either reflect individual variation among cells, or cell cycle dependence. Cell staining by P3490 was completely inhibited by its pre-incubation with the pS137-peptide, but was unaffected by the unphosphorylated S137-peptide (Fig. 1C). This contrasted the results on WB, and suggested that P3490 is highly phospho-specific when used for *in situ* cell staining, in which the pan-antibodies are nonreactive. In serum-starved NIH 3T3 cells, the RhoA activator lysophosphatidic acid (LPA) markedly increased the number of P3490-positive cells, and this was blocked by ROCK inhibition with Y-27632 (Fig. 1D). Hydroxyfasudil (OH-fasudil), a ROCK inhibitor structurally distinct from Y-27632, also dose-dependently reduced P3490 staining. These effects were visible microscopically (Fig. 1E), and easily quantified using a fluorescence plate reader following incubation with a fluorescently-labeled (Alexa Fluor®488) secondary antibody (Fig. 1F). At 50 µM, OH-fasudil eliminated P3490 staining in nearly all cells without affecting their total PFN1 levels (Fig. 1E).

**Figure 2:**
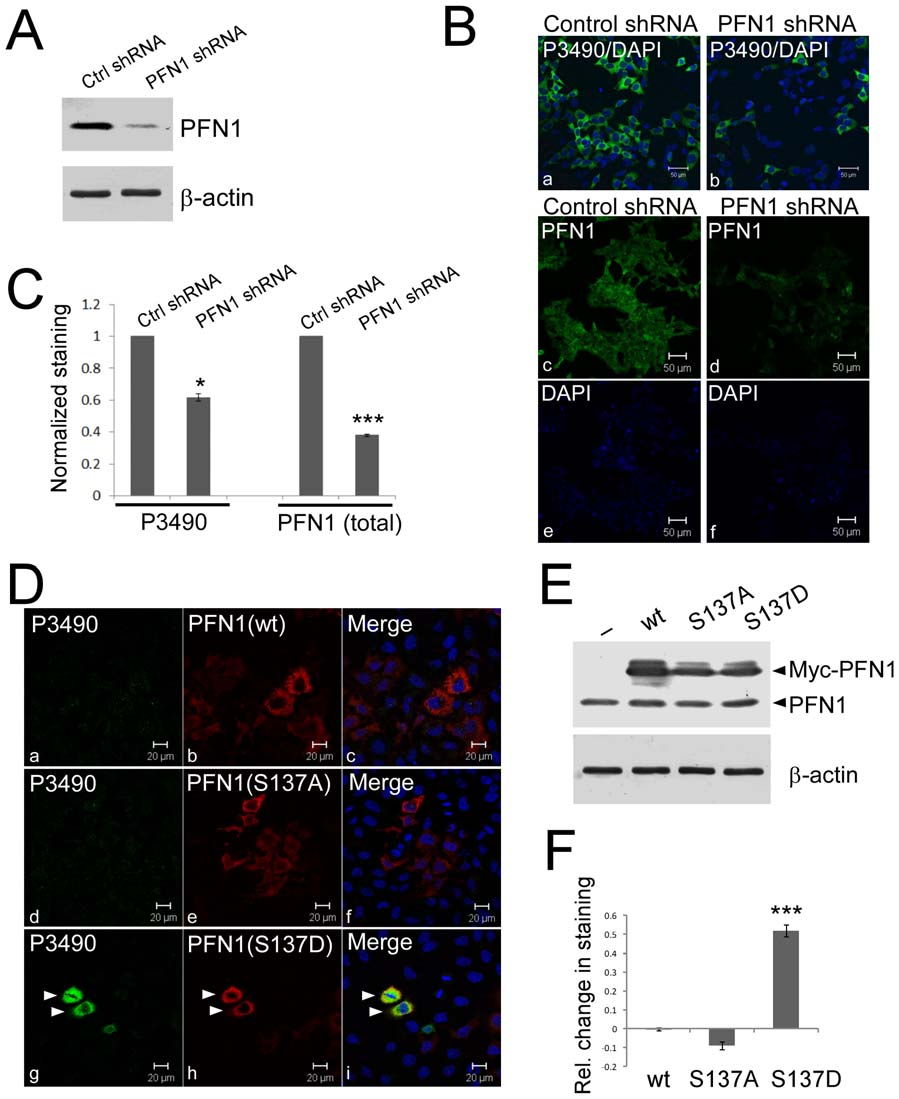
P3490 specifically detects pS137-PFN1 via immunofluorescence staining. *A*, HEK293 cells were infected for three days with lentiviral shRNA particles encoding either a scrambled nucleotide sequence (Ctrl shRNA), or a target-specific sequence for human PFN1 (PFN1 shRNA). Efficient PFN1 knockdown was confirmed by Western blot. *B*, virus-mediated PFN1 knockdown reduced P3490 staining (green, a–b), as visualized by confocal fluorescence microscope. Total PFN1 decrease was also confirmed by immunofluorescence staining (green, c–d). DAPI (blue) was used to counterstain cells either as merged (a–b) or unmerged images (e–f). *C*, staining of virus-infected cells with P3490 and PFN1 antibodies (as in A and B) was quantified by fluorescence plate reader, and normalized vs. DAPI to control for cell numbers. The decrease of P3490 (∼40%; *, p<0.05, unpaired t-test) and total PFN1(60%; p<0.001, unpaired t-test) staining caused by PFN1 knockdown was calculated relative to cells infected with the control shRNA (arbitrarily set as 1). Error bars represent the standard error of the mean (SEM). Data are mean ± SEM of three independent experiments. *D*, HEK293 cells were transiently transfected with Myc-tagged PFN1(wt, S137A or S137D), treated with 50 µM hydroxyfasudil for 16 hr, and double stained with an anti-Myc (red) and P3490 (green) antibodies. Only cells expressing the phosphomimetic Myc-PFN1(S137D) stained positive with P3490 (g–i), but not Myc-PFN1(wt) (a–c) or Myc-PFN1(S137A) (d–f). Arrowheads indicate two cells that expressed Myc-PFN1(S137D) and stained positive with P3490. *E*, Western blot confirmed comparable levels of Myc-PFN1 over-expression (wt, S137A and S137D) in transiently transfected HEK293 cells, as compared to cells transfected with an empty vector (pcDNA3). *F*, cells transfected (as in E) with pcDNA3 or various PFN1 constructs were immunostained with P3490, and quantified on a fluorescence plate reader with normalization vs. DAPI. The effects of PFN1 over-expression on P3490 staining are represented as relative change compared to cells transfected with pcDNA3. Only PFN1(S137D) increased P3490 staining (***, p<0.001, unpaired t-test). Cells were not treated with hydroxyfasudil. Error bars represent the SEM. Data are mean ± SEM of three independent experiments.

We further confirmed the specificity of P3490 for pS137-PFN1 using RNAi knockdown of endogenous PFN1. HEK293 cells were transduced with lentiviral shRNAs targeting PFN1 (Fig. 2A), which reduced P3490 staining (Fig. 2B–C), consistent with PFN1 being the cellular target of the antibody. This effect was evident microscopically (Fig. 2B), and was quantified using the fluorescence plate reader (Fig. 2C). The relative decrease in total PFN1 level (60%) as a result of shRNA knockdown was larger than that of P3490 staining (40%). This implies that Ser-137 phosphorylation of PFN1 may need to be kept at a certain level in the cell, and could be regulated in a fashion partially independent of total PFN1 levels. In addition, P3490 stained ectopically expressed phosphomimetic PFN1(S137D) in cultured cells, but not PFN1(wt) or PFN1(S137A). This was most evident when phosphorylation of endogenous PFN1 at Ser-137 was inhibited by OH-fasudil (Fig. 2D), and was also quantified by the fluorescence plate reader (Fig. 2E and F). Taken together, these results confirmed the specificity of P3490 for pS137-PFN1 for *in situ* staining.

### Phosphatase inhibitors increase pS137-PFN1 levels in cultured cells

**Figure 3:**
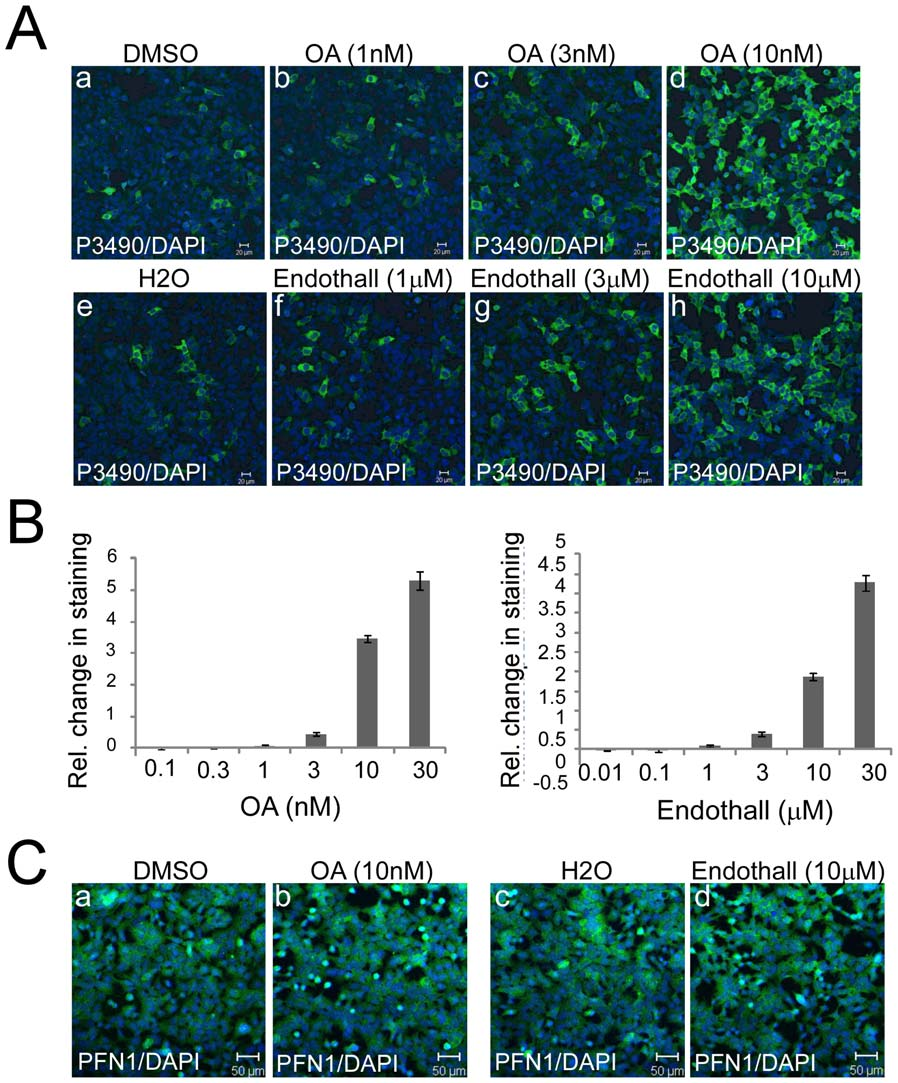
Pharmacologic inhibition of serine/threonine phosphatases increases pS137-PFN1 in cultured cells. *A*, HEK293 cells were treated with okadaic acid (OA) (a–d) or endothall (e–h) for 16 hr at increasing concentrations (1, 3 and 10 nM for OA; 1, 3 and 10 µM for endothall), followed by immunostaining with P3490 (green) and counterstaining with DAPI (blue). The number of P3490-positive cells and overall P3490 staining intensity increased notably at 10 nM of OA and 10 µM of endothall treatment. *B*, effects of OA and endothall on P3490 staining were quantified using a fluorescence plate reader. P3490 levels were normalized vs. DAPI, and the effects of the drugs were represented as relative change vs. the vehicle controls. OA and endothall increased P3490 staining dose-dependently. Error bars represent the SEM. Data are mean ± SEM of three independent experiments. *C*, HEK293 cells treated with 10 nM OA (a–b) or 10 µM endothall (c–d) were immunostained for total PFN1 (green) and counterstained with DAPI (blue). Merged images showed no changes in total PFN1 levels as a result of the drugs.

To identify candidate phosphatases for pSer-137 of PFN1, we employed pharmacologic inhibitors of serine/threonine phosphatases in cultured cells. Okadaic acid (OA) inhibits the serine/threonine-specific phosphoprotein phosphatase (PPP) family, in particular its two most abundant members, PP1 and PP2A [15]. Differential potencies of OA towards its target phosphatases have enabled its use to implicate candidates, particularly PP1 (IC_50_∼15–50 nM) and PP2A (IC_50_∼0.1–0.3 nM) [16]. Thus, we treated HEK293 cells with increasing concentrations of OA, and tested the effects on pS137-PFN1 levels by immunostaining with P3490. 16 hr of OA treatment markedly increased the number of P3490-positive cells at mid-nanomolar concentrations (≥10 nM), which was evident both microscopically (Fig. 3A) and after quantification using a fluorescence plate reader (Fig. 3B). The effective concentration of OA to increase pS137-PFN1 levels was much higher than its IC_50_ for PP2A (0.1–0.3 nM), and more consistent with its known IC_50_ for PP1 (15–50 nM). We also used endothall, a structurally distinct phosphatase inhibitor that is also much more potent against PP2A (IC_50_ = 90 nM) than PP1 (IC_50_ = 5 µM) [17], [18]. Like OA, endothall increased P3490 staining at concentrations (mid-micromolar) that are substantially higher than its IC_50_ for PP2A, but similar to that for PP1 (Fig. 3A and 3B). Due to cytotoxic effects at high concentrations, we were unable to carry out complete dose-responses of OA and endothall with regard to pS137-PFN1 levels. At their effective concentrations, neither drug affected the total PFN1 levels within cells (Fig. 3C). Fostriecin, a highly specific and potent inhibitor of PP2A (IC_50_ for PP2A = 1.5–5.5 nM; IC_50_ for PP1 = 45–58 µM), had no effect on P3490 staining, even at 1 µM (data not shown), a concentration expected to fully inhibit PP2A in a living cell [16]. Taken together, these results suggested that PP1, rather than PP2A, might be responsible for dephosphorylation of PFN1 at Ser-137.

### Genetic modulation of PP1 activity controls pS137-PFN1 levels in cultured cells

**Figure 4:**
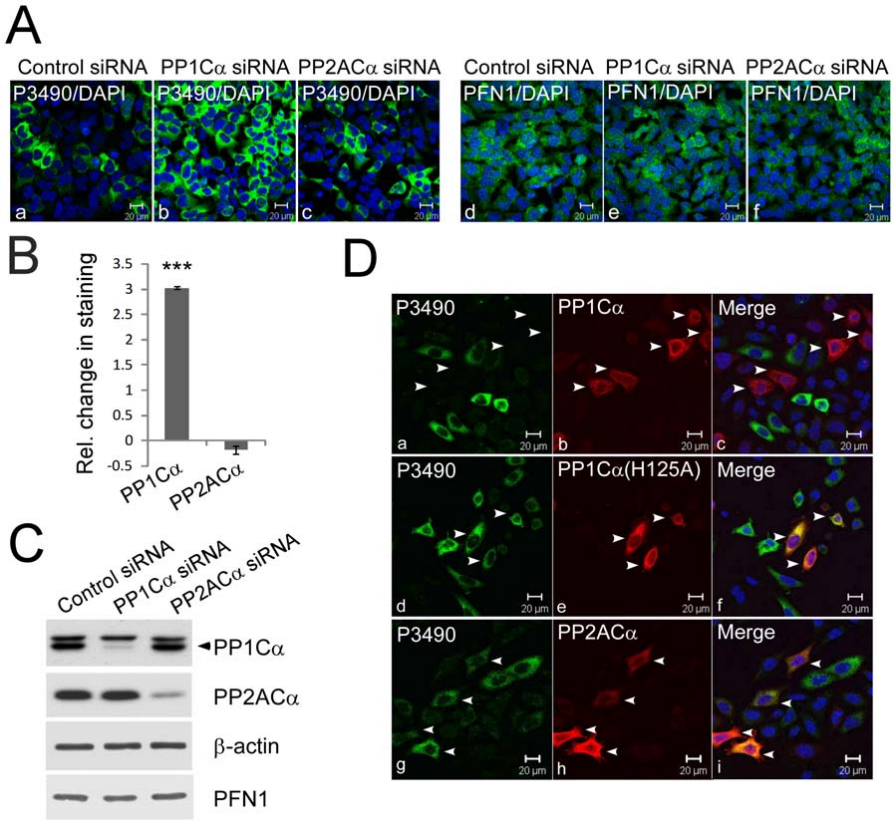
Protein phosphatase-1 (PP1) dephosphorylates PFN1 at Ser-137. *A*, HEK293 cells were transfected with control, or sequence-specific siRNAs targeting human PP1Cα or PP2ACα. They were immunostained with P3490 (a–c) or the antibody against total PFN1 (d–f) (both in green) and counterstained with DAPI (blue). Silencing PP1Cα, but not PP2ACα, increased the number of P3490-positive cells and their staining intensities, while having no effect on total PFN1 levels. *B*, effects of PP1Cα and PP2ACα knockdown on P3490 staining were quantified using the fluorescence plate reader, normalized vs. DAPI, and represented as change relative to control siRNA. PP1Cα knockdown increased P3490 staining three-fold (***, p<0.001, unpaired t-test), while PP2ACα knockdown had no effect. Error bars represent the SEM. Data are mean ± S.E.M. of three independent experiments. *C*, Western blot confirmed >90% knockdown of both PP1Cα and PP2ACα in HEK293 cells. Neither affected PFN1 levels. Arrowhead indicates PP1Cα. The identity of the protein band above PP1Cα, which cross-reacts with PP1Cα antibody, is unknown. *D*, HeLa cells were transfected with Myc-PP1Cα(wt) (a–c), Myc-PP1Cα(H125A) (catalytically inactive) (d–f) or Myc-PP2ACα (g–i), double immunolabeled with an anti-Myc antibody (red) and P3490 (green), and counterstained with DAPI (blue). Neither Myc-PP1Cα(H125A) nor Myc-PP2ACα over-expression affected P3490 staining of the transfected cells (as indicated by the presence of cells that were both red and green). However, PP1Cα(wt) over-expression inhibited P3490 staining (as indicated by the mutual exclusivity of red and green cells).

The above effects of phosphatase inhibitors indicated a role of PP1 in pS137-PFN1 dephosphorylation. To test this more definitively, we knocked down the alpha isoform of the PP1 catalytic subunit, PP1Cα, by transfecting HEK293 cells with siRNAs. In parallel, we also knocked down the alpha isoform of the PP2A catalytic subunit, PP2ACα. Based on Western blot analysis, endogenous PP1Cα and PP2ACα were both knocked down by 90% with no effect on cellular PFN1 levels (Fig. 4C). This was also confirmed via immunofluorescence staining of the transfected cells (Fig. 4A). We detected a three-fold increase of P3490 staining in cells transfected with the PP1Cα-specific siRNA in comparison to those transfected with the control siRNA (Fig. 4A and B). In contrast, silencing PP2ACα had no effect on P3490 staining (Fig. 4A and B). As an additional test, in HeLa cells we over-expressed wild-type PP1Cα, a catalytically inactive PP1Cα(H125A) mutant, or wild-type PP2ACα, and stained with P3490. Nearly all cells expressing wild type PP1Cα stained negative for pS137-PFN1, while many cells expressing the inactive PP1Cα(H125A) mutant or wild type PP2ACα stained positive for pS137-PFN1 (Fig. 4D). These data strongly suggested that PP1 dephosphorylates PFN1 at Ser-137.

### PP1 interacts with PFN1 in a pS137-dependent manner

**Figure 5:**
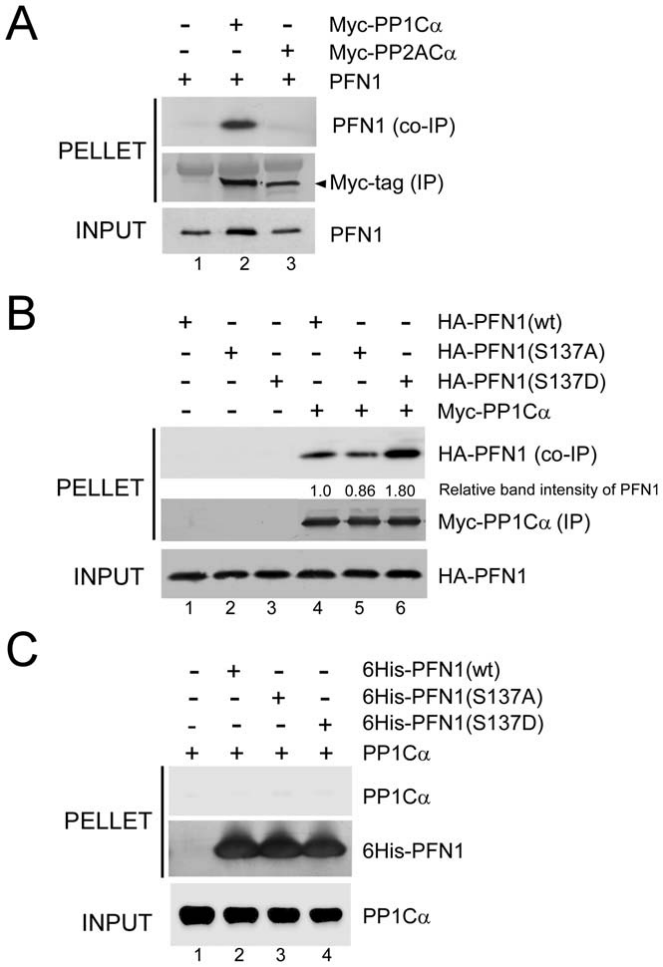
PP1 and PFN1 interact in cultured cells. *A*, HEK293 cells were co-transfected with an untagged PFN1 along with either an empty vector (pcDNA3) (Lane 1), or Myc-tagged PP1Cα (Lane 2), or PP2ACα (Lane 3), followed by immunoprecipitation with an anti-Myc antibody and Western blot for PFN1. PFN1 co-immunoprecipitated specifically with PP1Cα, but not PP2ACα. *B*, HA-PFN1 constructs (wt, S137A or S137D) were co-transfected into HEK293 cells with pcDNA3 (Lanes 1–3) or Myc-PP1Cα (Lanes 4–6). Myc-PP1Cα was immunoprecipitated with an anti-Myc antibody, and co-immunoprecipitated HA-PFN1 was detected by an anti-HA antibody. Relative to PFN1(wt), more PFN1(S137D) and less PFN1(S137A) bound to PP1Cα. *C*, Purified 6His-PFN1 (wt, S137A or S137D) proteins were bound to Ni-NTA beads, and subsequently mixed with recombinant PP1Cα (Lanes 2–4). Beads without His-PFN1 were mixed with the same amount of PP1Cα to control for nonspecific binding (Lane 1). Beads were washed, and analyzed for binding of PP1Cα to 6His-PFN1 by Western blot. 6His-PFN1 proteins were visualized by Coomassie blue staining. No specific binding was observed between purified PP1Cα and 6His-PFN1 (wt, S137A or S137D), despite that they were abundantly present.

The preceding experiments correlated PP1 activity with pS137-PFN1 dephosphorylation, but left uncertain whether the dephosphorylation is carried out directly by PP1, or by an unknown phosphatase whose activity is regulated by PP1. To test this, we transiently expressed Myc-PP1Cα and PFN1 in HEK293 cells, immunoprecipitated Myc-PP1Cα via an anti-Myc antibody, and tested for co-precipitation of PFN1. Myc-PP2ACα was separately co-expressed with PFN1 and immunoprecipitated as a control. We readily detected PFN1 in a complex with PP1Cα, but not PP2ACα (Fig. 5A). We next tested for preferential binding of PP1Cα to pS137-PFN1 vs. unphosphorylated PFN1 by using the phosphomimetic PFN1(S137D) vs. phospho-resistant PFN1(S137A) mutant. We transiently expressed HA-tagged PFN1(wt), PFN1(S137A) or PFN1(S137D) in HEK293 cells along with Myc-PP1Cα, followed by immunoprecipitation of Myc-PP1Cα and Western blot for co-precipitation of HA-PFN1. PP1Cα bound more phosphomimetic PFN1(S137D) and less phospho-resistant PFN1(S137A) in comparison to PFN1(wt), with all PFN1 variants expressed at a similar level (Fig. 5B). These data suggested that Ser-137 phosphorylation promotes PFN1 interaction with PP1, which subsequently dephosphorylates pSer-137.

PP1 is known to interact directly with a small number of substrates [19]. To determine if PP1 directly binds PFN1, we tested for their binding *in vitro*. We immobilized recombinant 6His-PFN1 (wt, S137A or S137D) proteins on nickel beads, mixed them with bacterially expressed recombinant PP1Cα, and subsequently tested for their interaction via Western blot against PP1Cα. We did not detect any specific binding of PP1Cα to all three forms of 6His-PFN1(wt, S137A and S137D), despite that all proteins were abundantly present (Fig. 5C). Thus, our data suggest that PP1 indirectly associates with PFN1 through an unknown linker protein, and dephosphorylates pSer-137.

## Discussion

Our prior studies were the first to demonstrate how PFN1's biological activities are regulated, at least in part, through phosphorylation at Ser-137 by the Rho-associated kinase ROCK. Ser-137 phosphorylation abolishes PFN1's binding to polyproline-containing ligands, e.g. huntingtin, which reduces its ability to inhibit polyglutamine-dependent huntingtin aggregation [6]. Our current work identifies PP1 as the principal phosphatase to dephosphorylate PFN1 at Ser-137. By using a phospho-antibody against pSer-137 of PFN1, we found that pharmacologic inhibition and genetic knockdown of the catalytic subunit of PP1 (PP1Cα), but not PP2A (PP2ACα), increased pS137-PFN1 levels in cultured cells. PP1Cα binds PFN1 in cultured cells. This interaction increases when Ser-137 is mutated to aspartate, mimicking phosphorylation, and decreases when it is mutated to alanine, blocking phosphorylation. The simplest interpretation of our data is that PP1 directly dephosphorylates pSer-137 of PFN1.

PP1 is a major eukaryotic serine/threonine phosphatase, and is thought to catalyze the majority of cellular protein dephosphorylation events [19]. There are four different catalytic subunits of PP1 in mammals (α, β, γ1,γ2), which are associated with a large number of regulatory subunits. This allows for hundreds of dimeric holoenzymes that are highly substrate-specific [19], [20], [21]. Our finding that PFN1 co-immunoprecipitates with PP1 from cultured cells in a phosphorylation-dependent manner suggests that they co-exist in a complex to dephosphorylate pSer-137. However, a lack of direct binding between these two proteins *in vitro* suggests that they are likely linked *in vivo* by an unknown PP1-interacting protein (PIP). This is consistent with the fact that PFN1 lacks a typical PP1-docking motif (RVxF, SILK or MyPhoNE (myosin phosphatase N-terminal element with the consensus sequence of RxxQV/I/LK/RxY/W)) that is found in most PIPs [19]. Given the vast number of PIPs estimated to be encoded by the human genome (∼650, 180 of which are known) [19], identifying the one specifically targeting PP1 to PFN1 could be a daunting task. However, screening PFN1-binding proteins for this PIP might be a more feasible approach. Our finding that mimicking Ser-137 phosphorylation (which should disrupt PFN1's binding to all polyproline-containing proteins) increases PFN1's interaction with PP1 argues against this PIP being a polyproline-containing ligand of PFN1. This effectively rules out the vast majority of PFN1-interacting proteins as the candidate, and focuses our future efforts on the limited number of non-polyproline ligands of PFN1 (e.g. actin and gephyrin) [3]. At a minimum, this will reveal the additional linker protein, if existent, between PFN1 and the unknown PIP, and take us one step closer to identifying it.

As an essential protein for all eukaryotic cells, PFN1 has now been linked to several human diseases such as Huntington disease [6] and cancer [7], [8], [9], [10], [11], [12]. It will thus be of great interest to identify therapeutic approaches to regulate its activity. Our prior and current identification of ROCK and PP1 as direct regulators of Ser-137 phosphorylation and dephosphorylation raises the possibility of targeting these two enzymes to manipulate PFN1 activity and treat these diseases. For example, ROCK inhibitors (e.g. fasudil), which are expected to up-regulate PFN1 activity, are already in clinical use to reduce vasospasm in the setting of subarachnoid hemorrhage [22], [23]. Importantly, they ameliorate behavioral deficits of HD transgenic mice [24] and have been validated as being anti-metastatic in several cancer models [25], [26], [27], [28]. Our work thus represents an early step in defining the mechanisms by which it may be possible to specifically control PFN1's activity to benefit patients.

## Materials and Methods

### Plasmids

Mammalian expression vectors encoding Myc-PP1Cα (wt or H125A) and Myc-PP2ACα were kindly provided by Dr. Hiroshi Shima (Miyagi Cancer Center Research Institute, Japan) [29]. Mammalian expression vectors encoding untagged or Myc-tagged PFN1 (wt, S137A and S137D) were cloned in pcDNA3 as described previously [6]. HA-tagged human PFN1 (wt, S137A and S137D) was PCR amplified and cloned into the EcoRI (5′) and XhoI (3′) sites of pcDNA3.1. Primer sequences are as follows: 5′-CCGGAATTCGCCGCCATGGCCTACCCATATGATGTTCCAGATTACGCTTCTTTGGGTGCCGGGTGGAACGCCTACATCGACAACCTCATG-3′ (upper primer containing an HA-tag); 5′-CCGCCGCTCGAGTCAGTACTGGGAACGCCGAAGGTGG-3′ (lower primer).

### siRNAs and shRNAs

Negative control siRNA (SI03650325) and human PP1Cα-specific siRNA (SI02225748) were purchased from Qiagen. A pool of three siRNAs targeting different regions of human PP2ACα were purchased from Santa Cruz (sc-43509). To silence PFN1, a 21nt sequence (5′-GGAATTTAGCATGGATCTTCG-3′) was custom-designed to target 246-267nt of human PFN1 mRNA, and cloned as short hairpin RNA (shRNA) downstream of the U6 promoter in a lentiviral vector, pFLRu-FH (kind gift of Dr. Greg Longmore) [30]. A control shRNA was similarly constructed using a non-targeting sequence (5′-CAACAAGATGAAGAGCACCAA-3′) adapted from the MISSION Non-Target shRNA control vector from Sigma. Lentiviral particles were purified by the Hope Center Viral Vectors Core at Washington University.

### Antibodies

Commercially available primary antibodies were as follows: rabbit anti-PFN1 (Cell Signaling, #3237), mouse anti-Myc tag (Santa Cruz, sc-40), rabbit anti-actin (Santa Cruz, sc-1616-R), rabbit anti-PP1Cα (Cell Signaling, #2582), rabbit anti-PP2ACα (Cell Signaling, #2038), mouse anti-HA tag (Covance, MMS-101P). Custom-made antibodies include rabbit polyclonal antibody against the C-terminus of PFN1 (KCYEMASHLRRSQY, gift of Dr. Nicholas A. DiProspero) and affinity purified rabbit polyclonal anti-pS137-PFN1 (New England Peptides, Ac-CMASHLRR(pS)QY-OH) [6]. Secondary antibodies used for Western blotting include alkaline phosphatase-conjugated secondary antibodies (Sigma, A3562) and horseradish peroxidase-conjugated secondary antibodies (Amersham Biosciences, NA9340V for anti-rabbit and NA931V for anti-mouse antibodies). Secondary antibodies for immunofluorescence staining include Alexa Fluor® 488-conjugated goat anti-rabbit IgG (Invitrogen, A-11034) and Alexa Fluor® 546-conjugated goat anti-mouse IgG (Invitrogen, A-11030). Agarose conjugated with secondary anti-mouse IgG (A6531) and EZview Red affinity gel conjugated with rabbit anti-c-Myc antibody (E6654) were purchased from Sigma.

### Reagents

Alpha isoform of bacterially expressed recombinant PP1 catalytic subunit was purchase from Sigma (P7937). Nickel-NTA beads were purchased from Qiagen (30410). Protease inhibitor cocktail (11-836-170-001) was purchased from Roche Diagnostics. ECL Plus Western blotting detection kit (RPN2132) was purchased from GE Healthcare. Lysophosphatidic acid (L7260) was purchased from Sigma. Okadaic acid (495609), Endothall (324760), Fostriecin (344280) and Hydroxyfasudil (390602) were purchased from Calbiochem. Fetal bovine serum (SH3007103) was purchased from Hyclone. Gold anti-fade mounting media was purchased from Invitrogen (P36934).

### Cell culture and transfection

HEK293, HeLa and NIH 3T3 cells were cultured in Dulbecco's modified Eagle medium containing 10% fetal bovine serum and penicillin/streptomycin. Lipofectamine 2000 (Invitrogen) was used for transfecting both DNAs and siRNAs using the protocol recommended by the manufacturer.

### Immunoprecipitation

HEK293 cells over-expressing Myc-PP1Cα(wt or H125A) or Myc-PP2ACα were lysed with buffer containing 50 mM Tris-HCl (pH 7.4), 150 mM NaCl, 0.1% Triton X-100, and protease inhibitors. Cleared lysate was mixed with agarose beads bound with anti-Myc antibody. After 2 hr of mixing at 4°C, beads were washed 4 times with lysis buffer, and analyzed by Western blot for co-immunoprecitation of untagged or HA-tagged PFN1 via antibodies against PFN1 (1∶1000) or the HA tag (1∶1000). For *in vitro* pull-down experiment, 20 µl nickel NTA beads were pre-blocked with 1% bovine serum albumin (BSA) for 30 min at room temperature, and subsequently mixed with 10 µg 6His-tagged PFN1(wt, S137A and S137D) proteins previously purified from bacteria [6] for 30 min at 4°C. After washing, 2 µg of recombinant PP1Cα was added to the beads in 100 µl binding buffer containing 10 mM Tris-HCl (pH 7.4), 150 mM NaCl, 0.1% BSA, 0.1% Triton X-100 and protease inhibitors. Following 1 hr incubation at 4°C, beads were washed 4 times with binding buffer without BSA, and analyzed by Western blot for PP1Cα or Coomassie blue staining for 6His-PFN1.

### Immunofluorescence staining

Cultured cells were fixed for 30 min with 4% paraformaldehyde, washed 3 times with PBS, followed by blocking with 2% BSA in PBS/0.1% Triton X-100 for 30 min. P3490 antibody was diluted at 1∶5000 (v/v) with blocking buffer and incubated with cells for 2 hr at room temperature or overnight at 4°C. Cells were washed 4 times with PBS/0.1% Triton X-100, incubated with 1∶500 dilution of Alexa Fluor® 488 goat anti-rabbit secondary antibody in blocking buffer for 2 hr at room temperature, washed 4 times with PBS/0.1% Triton X-100, and mounted. For total PFN1 staining, custom-made rabbit polyclonal antibody against the C-terminus of PFN1 (KCYEMASHLRRSQY) was used at 1∶100 and followed by secondary antibody incubation as for P3490. For double labeling, cells were simultaneously incubated with P3490 (1∶5000) and anti-Myc antibody (1∶1000) overnight at 4°C, followed by 2 hr incubation with 1∶500 dilution of Alexa Fluor® 488 goat anti-rabbit antibody and Alexa Fluor® 546 goat anti-mouse antibodies at room temperature. In all cases, DAPI was included in the secondary antibody solution at a concentration of 1 µg/ml. Images were acquired using the Zeiss LSM 5 PASCAL system equipped with the following lasers: 405 nm, 488 nm, 543 nm.

For quantitative immunostaining within 96-well plates, HEK293 cells were grown to approximately 90% confluence, fixed, blocked, stained with P3490 (1∶5000) or anti-PFN1 antibody (1∶100) and Alexa Fluor® 488 goat anti-rabbit antibody (1∶500), and counterstained with DAPI as described above. Cells were washed 4 times with PBS/0.1% Triton X-100, left in 150 µl PBS, and quantified for fluorescence intensity (490/519 nm for Alexa Fluor® 488 and 365/439 nm for DAPI) using a fluorescence plate reader (INFINITE M1000, Tecan, Inc). Background fluorescence was acquired using cells stained only with the secondary antibody. Background subtracted fluorescence intensity for Alexa Fluor® 488, which represented P3490 reactivity, was then normalized against DAPI level to control for total cell number. For all experiments, at least 4 replicate wells were quantified for a given condition, and each experiment was repeated at least three times independently.

### Antigen competition

P3490 was first diluted 1∶5000 in the blocking buffers for immunofluorescence (PBS/0.1% Triton X-100/2% BSA) or Western blot (TBS/0.05% Tween 20/5% skim milk), and then mixed at 1∶200 molar ratio (antibody/peptide) with PFN1 peptides containing (Ac-CMASHLRR(pS)QY-OH) or lacking a phosphate on Ser-137 (Ac-CMASHLRRSQY-OH) for 30 min at room temperature. A control antibody solution was set up by mixing an equivalent volume of water (instead of peptides) with P3490. All antibody solutions (no peptide, S137-peptide and pS137-peptide) were subsequently used to immunostain or immunoblot HEK293 cells as described above.
